# Underground Ecosystem Conservation Through High-resolution Air Monitoring

**DOI:** 10.1007/s00267-022-01603-0

**Published:** 2022-02-21

**Authors:** Rosangela Addesso, Alessandro Bellino, Daniela Baldantoni

**Affiliations:** Department of Chemistry and Biology “Adolfo Zambelli”, University of Salerno, Via Giovanni Paolo II, 132, 84084 Fisciano (SA), Italy

**Keywords:** Atmospheric monitoring, Frequency spectra, Show caves, Tourist load, Time series

## Abstract

In cave ecosystems tourists represent moving sources of discontinuous disturbances, able to induce transient system responses whose knowledge is crucial in defining appropriate conservation measures. Here we propose an approach to evaluate the amplitude and scales of cave alterations based on high-resolution air monitoring, through the use of purposely developed low-cost monitoring stations and a consistent analytical framework for information retrieval based on time series analysis. In particular, monitoring stations adopt a modular structure based on physical computing platforms acquiring data through several sensors, with means of preventing humidity damages and guaranteeing their continuous operation. Data are then analyzed using wavelet periodograms and cross-periodograms to extract the scales of tourism-induced alterations. The approach has been exemplified in the Pertosa-Auletta Cave, one of the most important underground environments in Southern Italy, highlighting the development of monitoring stations and the information obtainable with the proposed analytical workflow. Here, 2 monitoring stations acquiring data for 1 year at 1′ sampling time on temperature, relative humidity, CO_2_, VOCs, and particulate matter were deployed in trails subjected to different levels of tourism. In terms of Pertosa-Auletta Cave air dynamics, the approach allowed estimating the temporal and spatial scales of tourism-induced alterations in the order of minutes and meters, respectively, with parameter-dependent variations. On more general terms, the approach proved reliable and effective, with its modularity and low-cost fostering its straightforward adoption in other underground ecosystems, where it can support the development of tailored management strategies.

## Introduction

Caves are one of the most fragile ecosystems on Earth (White 2019). Being confined places, characterized by little spatial and temporal variability of several ecological factors, every anthropogenic disturbance can easily trigger alterations taking longer to settle, and possibly never returning to the initial states (Lobo et al. 2013). Unfortunately, the wonders of these peculiar ecosystems attract thousands of visitors every year (de Freitas 2010, Lobo et al. 2015; Danardono et al. 2018), that represent a serious threat for their preservation. The understanding of the nature and extent in space and time of the anthropogenic disturbances in caves is thus a priority for their sustainable management and long-term conservation (Cigna and Burri 2000; Korzystka et al. 2011; Lobo et al. 2013, 2015).

Cave atmosphere is particularly affected by tourism through processes including the release of body heat, water vapor and CO_2_, as well as the introduction (e.g. through spores) of allochthonous species (Chiesi 2002; Russell and MacLean 2008; Smith et al. 2013). All of these processes are able to affect the reactions at the interface between atmosphere, lithological substrate, water film and microbial community, that in turn controls most ecosystem processes (Pulido-Bosch et al. 1997; Chiesi 2002; Calaforra et al. 2003; Milanolo and Gabrovšek 2009; de Freitas 2010; Lang et al. 2015a, b; Carrasco et al. 2016; De Vincenzi et al. 2016; Howarth 2019; Addesso et al. 2022).

Apart from tourist load in terms of number of tourists as well as length and frequency of the visits, the degree of cave vulnerability to tourism depends on its age, morphology, substrate and hydrological characteristics. For example, morphological characteristics can produce internal climatic zonation, creating several spatial and temporal microclimatic niches that affect cave responses to changes (Russell and MacLean 2008).

Bringing all these aspects together in defining successful policies and management practices is a challenging task. In this context, the adaptive ecosystem management paradigm, considered the gold standard in addressing these challenges, pivots on environmental monitoring as the process allowing gathering information from the environment and assessing action outcomes, promoting their continuous adaptation. Although the natural dynamics, either spatial or temporal within the cave ecosystems usually occur at large scales (meters/hours and above), tourism-induced alterations can manifest themselves in transients at finer scales. This is especially true when considering the effects of tourism on the cave atmosphere, characterized by a naturally higher variability in space and time as compared to other environmental compartments. The understanding of the pressure exerted on the cave ecosystem by anthropogenic activities requires thus high-resolution monitoring, in either space or time, that can be financially and technically challenging. Developing novel robust and low-cost means to continuously monitor the cave atmosphere can be thus crucial in empowering managers with the right tools to apply the adaptive ecosystem management to these systems. However, the monitoring itself proves pointless without the proper means of aggregating and analyzing the amount of data produced. In this context, the integration of advanced time series analysis with high-resolution monitoring has the potential to provide insightful results, through the extraction of ecologically meaningful information on small-scale transients within the system.

Moving from these considerations, the present research aimed at integrating high time resolution monitoring within a consistent analytical framework focused on evaluating the effects of anthropogenic alterations, their spatial behavior and their temporal scales. The implementation relied on the development of novel robust and low-cost monitoring stations able to collect data on a large number of atmospheric parameters, and on the analysis of their temporal variations on sub-hourly to monthly scales. The approach has been exemplified through a field study in the Pertosa-Auletta Cave (Campania region, Italy), one of the largest underground systems of Southern Italy (Addesso et al. 2019, 2021), hosting more than 60.000 visitors per year. The research was able to exploit also the unique scenario set up by the SARS-CoV-2 outbreak, creating an involuntary experimental setting, allowing evaluation of effects of different cave management policies.

## Materials and Methods

**Fig. 1:**
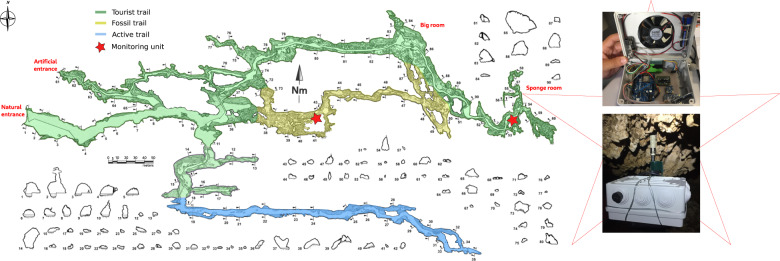
The Pertosa-Auletta Cave system, with indication of the sectional profiles, the natural (always open, but inaccessible to tourists) and artificial (open on-purpose for tourist transit) entrances, the main trails (in different colors), and the locations of the monitoring stations (red stars). Images of the stations with the internal electronics and installed in situ are also shown

The continuous monitoring of key atmospheric parameters (temperature, relative humidity, pressure, CO_2_, VOC, particulate matter) was carried out through the development of two monitoring stations focused on providing good accuracy, robustness toward prolonged operation in saturating relative humidity and low cost. In particular, the stations were built around the popular ESP8266EX (Wemos D1 R2 development board, China) featuring a Tensilica L106 32-bit microprocessor with 80 MHz (160 MHz max) clock and a Wi-Fi module. Sensors for temperature, relative humidity, pressure (not discussed in the present research), CO_2_, VOC, typical particulate size (weighted average of the particle sizes in the analyzed air volume), PM0.5, PM1.0, PM2.5, PM4.0, and PM10, the details or which are reported in Table S1, were operated through a single I2C bus for the digital readout. In order to provide a more general expression of particulate matter concentration, data were expressed as mass per air volume, forgoing PM0.5 data measured by the SPS30 sensor as number of particles per air volume only. Data retrieved were written at each cycle on a microSD card, by means of an I/O board operated through an SPI interface. A high accuracy (±2 ppm) DS3231 real-time clock (Maxim Integrated, USA) was included in the design and operated through the I2C bus. The entire system was powered through a switching power supply operated in SEPIC mode (Torpedo, Futura Elettronica, Italy), allowing the seamless switch between DC (5 V) and a LiPo battery granting ~10 h of operation, recharged when DC was available. The entire electronics were enclosed in standard electrical cassettes and mounted upside-down (Fig. 1), with a fan continuously pulling air from below the stations and pushing it inside the cassette. Such a design allowed avoiding the occurrence of condensation upon the electronics and their long-term flawless operation. All the sensors were built inside the cassettes, with the exception of one of the temperature/relative humidity sensors (SH10/Adafruit 1298) enclosed in a sintered aluminum case, allowing its operation at ambient conditions and avoiding biased readings due to the heat from electronics. The presence of redundant sensors (3 for temperature and 2 for relative humidity) increased the robustness of the apparatus, allowing the crossed check of sensor functioning. Moreover, a push notification system was implemented within the board software through the Pushetta API (https://www.pushetta.com), allowing sending real-time notifications about possible malfunctioning of the stations on smart devices through the Wi-Fi module.

The two monitoring stations were located in two sections of the Pertosa-Auletta Cave (Fig. 1), hereafter referred as “tourist trail” and “fossil trail” according to the official denomination of the managers, the description of which is extensively reported in Addesso et al. (2019, 2021, 2022). Station positioning aimed at evaluating the relative amplitude and scales of the tourist-induced contributions upon the natural fluctuations in atmospheric parameters, allegedly mainly driven by the cave coupling with the external environment. As such, the stations were set up in places subjected to different levels of tourism and at increasing distance from the cave natural entrance (Fig. 1). In particular, the first one was installed in the final part of the tourist trail, which is open to the public, lit, and rich in speleothems due to the intense dripping activity. The second one was installed in the middle of the fossil trail, which is closed to the public to protect several bats colonies, unlit, with few speleothems, numerous fractures, and collapsing deposits. In this way, the latter should be able to record the natural fluctuations in atmospheric parameters with little contribution from tourists. Such contribution should instead be recorded with high sensitivity by the former, due to the proximity of tourist transit and the expected lower background fluctuations in the innermost part of the cave.

Data were collected every minute from August 2019 to September 2020, with a brief (2 weeks) interruption on January 2020 due to technical problems with the cave power supply. All of the parameters were acquired during the entire period, with the exception of CO_2_, whose data were missed from November 2019 to January 2020 due to an erroneous sensor recalibration that was later fixed. Overall, the monitoring period consisted of 3 phases defined by the different tourism management: i) unrestricted tourism (1 August 2019 to 9 March 2020), when an unlimited number of tourists joined tours to visit the cave, ii) pandemic-related lockdown (10 March 2020 to 10 June 2020), when the cave gates were closed and nobody was allowed entering the cave, and iii) controlled tourism (11 June 2020 to 1 September 2020), when fewer and smaller tourist groups were allowed visiting the cave at the same time, with shorter tours. Moreover, the path followed by tourists during the 3^rd^ phase ended in the “Big room” instead of the “Sponge room” (Fig. 1), preventing tourists reaching the section monitored by the station in the tourist trail.

The monitoring station output consisted of a CSV file format, with data organized in a matrix structure, and the (UTC) time-date field complying with the ISO 8601 format. Data were processed and analyzed within the R 4.10 programming environment (R Core Team 2021), with functions of the “lubridate”, “tsbox”, “tsibble”, “fable”, “feasts”, “anomalize”, and “WaveletComp”. In particular, data were imported and represented as multivariate time series using the *tsibble* object representation. Data on the number of visitors within the cave, derived from the manager registers, were expressed on the same time base of the monitoring data and added to the latter, in order to evaluate possible relationships between tourist load and atmospheric parameters. Time series were individually cleaned from outliers, identified on the residuals of STL seasonal decomposition models through the interquartile range method, and substituted by values estimated from the STL models. In order to estimate the multiple temporal scales subtending the fluctuations in the monitored parameters and evaluate the possible co-variations between them and the tourist load, wavelet periodograms and cross-periodograms were calculated. In particular, the frequency spectrum of time series was analyzed through Morlet wavelet transformation and the significance of the periods was evaluated through simulations (*n* = 10) against white noise. The phase information derived from wavelet analysis was employed instead in the analysis of the co-variations of bivariate time series, through the calculation of wavelet cross-power spectra.

## Results

**Fig. 2:**
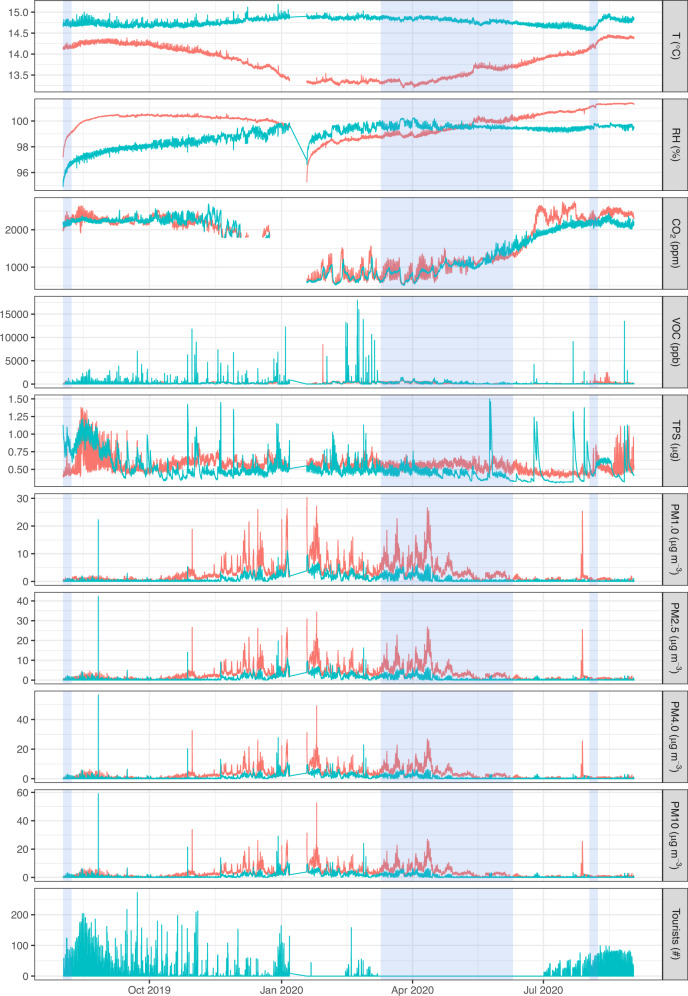
Time series of all the parameters monitored (temperature: T, relative humidity: RH, CO_2_, VOC, typical particulate size: TPS, PM1.0, PM2.5, PM4.0, PM10) in the tourist (green) and fossil (red) trails, as well as the number of tourists in the cave. Shaded areas indicate, in the order, the portion of time series detailed in Figs. S2 (extract from the phase of unrestricted tourism), S1 (pandemic-related lockdown) and S3 (extract from the phase of controlled tourism)

The dynamics of all the analyzed parameters over the monitoring period are shown in Fig. 2, with the monthly minima and maxima reported in Table S2. Temperature, relative humidity, and CO_2_ are the only ones showing large-scale temporal dynamics, all the others appearing stationary over the year of monitoring, with variations mostly occurring at small scales. These variations span several orders of magnitude over the baseline in the case of VOCs and particulate concentrations or are contained within three times the baseline in the case of the typical particulate size. In the case of particulate concentrations, the widest fluctuations occur between November and June, whereas the largest VOC fluctuations are evenly distributed across the year of monitoring, with the exception of the period from March to July (Fig. 2). The redundant temperature and humidity sensors provided the same trends of the SHT10 sensor enclosed in the sintered aluminum case, with 0-lag cross-correlations *r* > 0.71 (*P* < 0.001) in all the cases.

The lockdown phase spanned almost 4 months and allows evaluating the background levels of the parameters with stationary fluctuations, by removing the contribution of anthropogenic activities within the cave. An excerpt of the dynamics recorded during this phase is shown in Fig. S1. Daily fluctuations are evident in most of the traces, especially in VOCs, with comparable oscillations in both the trails, CO_2_ monitored in the fossil trail and, to a lesser extent, relative humidity in the tourist trail. Daily oscillations are recorded also in particulate matter concentrations, with the different dimensional classes sharing the same trend, superimposed on faint weekly fluctuations in the case of the fossil trail. In all the traces, the amplitude of the variations reduces from the onset May. The typical particulate size is unique among the parameters investigated in showing approximately constant values over the entire period, which are comparable to the limit of detection of the sensor employed (~0.3 particles cm^−3^). On the opposite, relative humidity is always close to the upper limits of sensor functioning, with a progressive increase in the fossil trail.

In terms of the dynamics during cave opening to the public, an excerpt from the first week of August in 2019 and 2020, under unrestricted and controlled tourism, respectively, is shown in Figs. S2 and S3. Daily oscillations are more evident in 2019 than in 2020, especially in CO_2_, typical particulate size and temperature, as well as in the tourist trail in respect to the fossil trail. Superimposed on the daily oscillations, CO_2_, temperature, VOCs and, to a lesser extent, relative humidity also show small-scale variations that are concurrent with the passage of tourists. These variations were recorded in 2019 and in the tourist trail only, with the exception of CO_2_, temperature and typical particulate size that show them also in the fossil trail or, in the case of the latter, exclusively in this trail. VOCs are the unique parameter showing small-scale variations following the passage of tourists in 2020, which are evident in the fossil trail only.

**Fig. 3:**
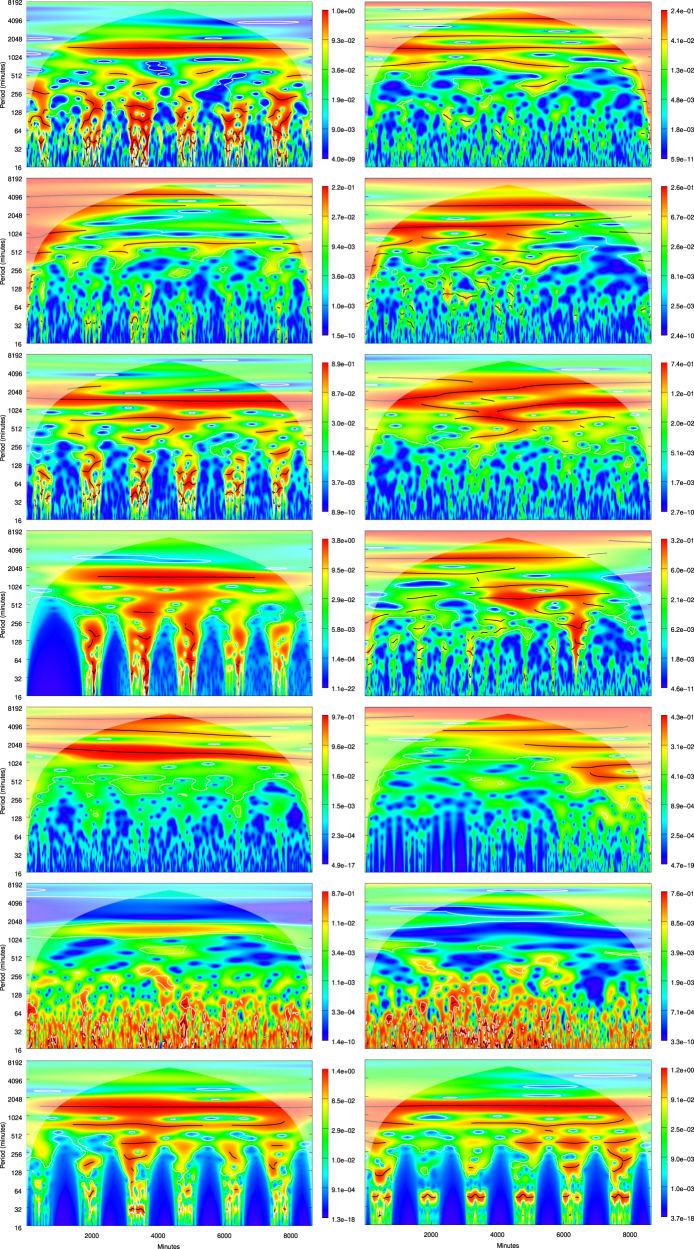
Wavelet periodograms of the time series (from top to bottom: temperature, relative humidity, CO_2_, VOC, typical particulate size, PM10, number of tourists) during the first week of August 2019 (left column - from 2019-08-02 04:41:00 to 2019-08-08 04:41:00) and of August 2020 (right column - from 2020-08-02 04:41:00 to 2020-08-08 04:41:00) in the tourist trail. The *x*-axis indicates the minutes from the beginning of the time series, whereas the *y*-axis the wavelet periods (in minutes). Due to the similarity among the time series relative to the particulate matter classes, only the periodogram for PM10, as representative of the others, is shown. The wavelet power spectrum is represented on quantile scales, with white lines enclosing regions of significant (for *α* = 0.05) periods and black lines indicating wavelet ridges

**Fig. 4:**
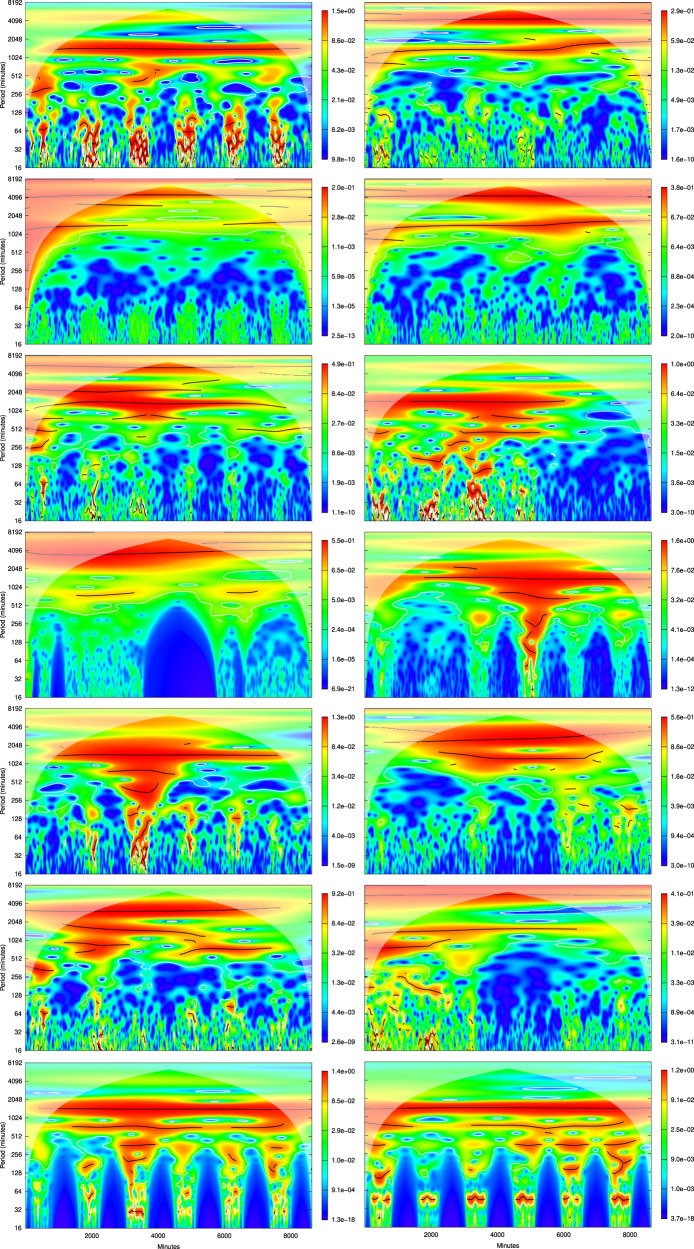
Wavelet periodograms of the time series (from top to bottom: temperature, relative humidity, CO_2_, VOC, typical particulate size, PM10, number of tourists) during the first week of August 2019 (left column - from 2019-08-02 04:41:00 to 2019-08-08 04:41:00) and of August 2020 (right column—from 2020-08-02 04:41:00 to 2020-08-08 04:41:00) in the fossil trail. The x-axis indicates the minutes from the beginning of the time series, whereas the y-axis the wavelet periods (in minutes). Due to the similarity among the time series relative to the particulate matter classes, only the periodogram for PM10, as representative of the others, is shown. The wavelet power spectrum is represented on quantile scales, with white lines enclosing regions of significant (for *α* = 0.05) periods and black lines indicating wavelet ridges

Periodograms for the parameters analyzed in the tourist (Fig. 3) and the fossil (Fig. 4) trails highlight the presence of daily oscillations in most of the traces with harmonics at both higher and lower periods. In the tourist trail, CO_2_, VOCs, temperature and, to a lesser extent, relative humidity show significant sub-day oscillation periods that are consistent with the passage of tourists in 2019, with the smallest ones in the order of a few minutes. Particulate matter also shows high-frequency oscillations, but evenly distributed over the time span of the analysis and with a reduced contribution of daily fluctuations, that primarily determine, instead, the time series of the typical particulate size. The high-frequency oscillations disappear in 2020 in the tourist trail from almost all the parameters but, where present, are either evenly distributed along the time series such as in particulate matter, or scattered throughout the time span of the analysis such as in relative humidity and VOCs, without clear patterns. With the exception of temperature, typical particulate size, and CO_2_, all the parameters recorded in the fossil trail in 2019 do not show high-frequency variations consistent with the passage of tourists or, when present, their patterns are less clear than those observed in the tourist trail, especially for the typical particulate size and CO_2_. The absence of frequency components attributable to the passage of tourists is common among the periodograms for the parameters recorded in the fossil trail in 2020, with the notable exception of VOCs.

**Fig. 5:**
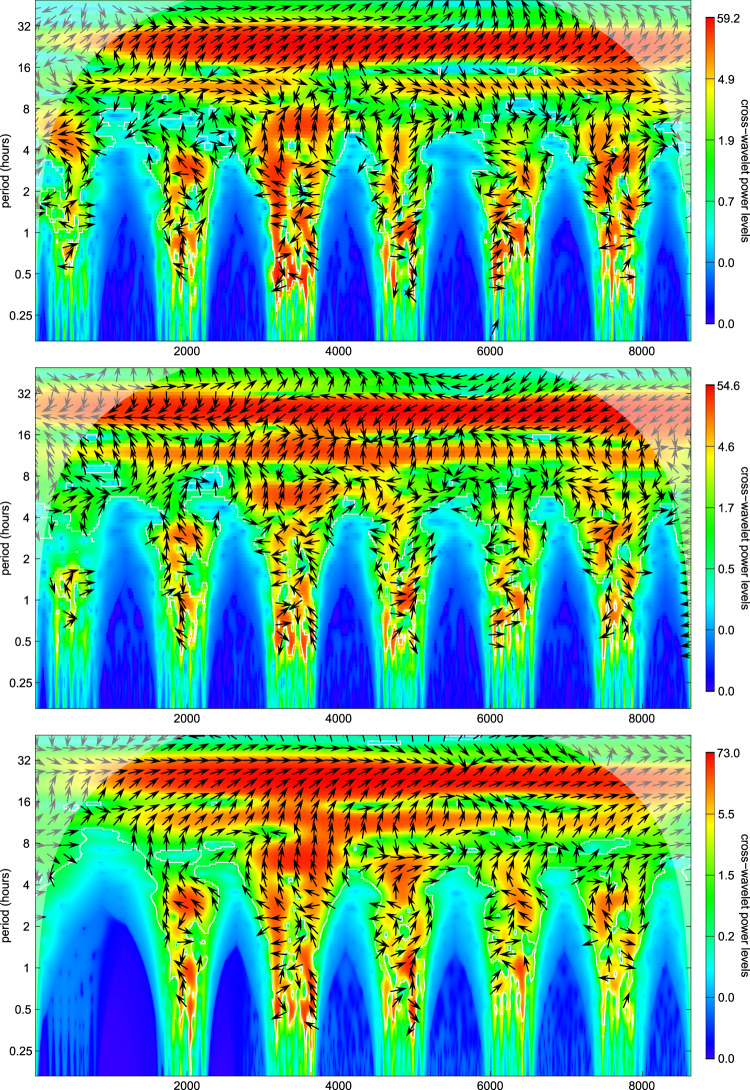
Wavelet cross-periodograms between temperature (upper panel), CO_2_ (middle panel), VOC (bottom panel), and the number of tourists during the first week of August 2019 (from 2019-08-02 04:41:00 to 2019-08-08 04:41:00) in the tourist trail. The x-axis indicates the minutes from the beginning of the time series, whereas the *y*-axis the wavelet periods (in hours). The cross wavelet power spectrum is represented on quantile scales, with white lines enclosing regions of significant (for *α* = 0.05) periods and black lines indicating wavelet ridges. Arrows represent the relative phase of the tourist and the parameter wavelets: wavelets are in-phase in I and IV quadrants and out-of-phase in II and III quadrants, with the tourist leading in the I and III quadrants and lagging in the II and IV. The arrow angle indicates the phase difference between the wavelets of the two series

The synchronicity between the time series of the monitored parameters and of the number of tourists was analyzed on the series showing the clearest signals, i.e., CO_2_, temperature, and VOCs recorded in the tourist trail in 2019 (Fig. 5). The daily oscillations are in-phase in the case of temperature and VOCs and out-of-phase in the case of CO_2_, with the number of tourists always representing the leading trace. The synchronicity in the harmonics at 12 h is similar, instead, between CO_2_ and VOCs, both in-phase and with the number of tourists as the leading trace, whereas it represents the lagging trace in the case of temperature. At periods lower than 12 h all the cross-periodograms show wide phase variations, similar among the parameters, on time scales in the order of minutes/few hours, with the number of tourists shifting from the leading to the lagging trace.

## Discussion

In terms of hardware, the monitoring stations proved effective and reliable over the entire experimental period, providing 1-minute resolution data for 13 months without clear evidences of long-term drifts or general electrical problems. The data from the redundant sensors further demonstrate the accuracy of the temperature and relative humidity recordings, a remarkable result considering the low cost of the stations, with raw prices in the order of one hundred euros. The unique fault observed during the experiment was on the software side, caused by a recalibration routine embedded within the CO_2_ sensor that was later disabled. In spite of the lack of issues, however, two technical limitations are worth mentioning. The first lies in the inability of the particulate sensor to provide reliable size-partitioning of the particulate matter, an issue attributable to the low particulate concentration and its typical size, with values near the lowest limits of detection of the SCD30 sensor employed. The second limitation involves the long burn-in phase of the relative humidity sensor, attributable to its enclosing in a sintered aluminum case, needed to prevent condensation from harming the sensor. Overall, however, although they slightly limited the availability or quality of data, neither of these issues represented a critical drawback of the stations for the aims of the present research.

In terms of system dynamics, the positioning of the monitoring stations and the different cave management adopted over the time created spatial and temporal references allowing evaluation of the cave natural dynamics, the effects of tourism, and how cave morphology shapes system responses. By all accounts, the effects of tourism appears negligible at large temporal and spatial scales, notwithstanding its intensity. Indeed, the strong seasonality in parameters like CO_2_ and temperature, with contrasting trends between autumn-winter and spring-summer, is consistent with the hypothesis of dynamics controlled by the annual variations in solar irradiation in the Northern hemisphere. The tourism does not appear to affect these trends, as demonstrated by the springtime increase in CO_2_ concentrations pre-dating the cave opening to the public and the lack of signal from the onset of tourism on the trends. These considerations apply to both the trails that, however, show interesting differences in their behavior regarding either the trends in the parameters analyzed or the amplitude of daily oscillations.

The former scenario involves the temperature, whose annual trends between the trails are in anti-phase, i.e. the fossil trail cools down while the tourist trail warms up. To an extent, a similar contrasting behavior between trails is appreciable also in relative humidity, although far less clearly due to the burn-in phase of the sensors and the values always close to saturation. The morphology of the cave (Addesso et al. 2019, 2021), with the fossil trail directly exposed to the inflow of external air from the big natural entrance (30 m × 70 m) and the end of the tourist trail constituting the recessed innermost part of the cave, may account for this behavior. On the one hand, it is certainly responsible for the reduced amplitude of the annual variations in the tourist trail but, on the other hand, may also explain its rise in temperature when a decrease was actually expected. Indeed, the cooling of the cave, beginning from the cave entrance, can force the water vapor in the innermost part of the cave to progressively condense and release latent heat. Such a hypothesis is in line with the described intertwined dynamics among external temperature, rock cooling/warming, and air drying/wetting (De Freitas and Littlejohn 1987; Forbes 1998; de Freitas 2010) and can account for the rise in temperature during winter-spring. Although the relative humidity trace in theory should provide insights on this process, the values always close to saturation and the continuous inputs of vapor associated to the water movements, especially intense in the tourist trail, actually hamper testing such a hypothesis.

The dampening effect of cave morphology on the temperature changes in its innermost part reverberates also on other parameters, especially particulate concentrations. Indeed, they show wider daily oscillations in the fossil trail than in the tourist trail, a behavior that can be explained by the influx of external air from the big entrance conveying particulate matter (Badino 2010). In this context, it is interesting to note hints of weekly fluctuations in particulate concentrations during the lockdown phase, indicating the coupling of these concentrations with what happens in the outer environment. Indeed, weekly signals in time series are signatures of anthropogenic effects that, in the case of the Pertosa-Auletta Cave, could be exerted either directly, e.g., through the influx of air affected by vehicular traffic, or indirectly through possible alterations of bat activity. However, the occurrence of higher particulate concentrations during winter-spring, a period of frequent windy and rainy weather in the area, suggests that the influx of external air primarily controls particulate dynamics within the cave. An important corollary result of these trends is the negligible effect exerted by tourists on particulate concentrations, with the lowest values observed during the highest tourist loads. This is obviously a result of the highly controlled behavior of tourists enforced by guides, but nonetheless suggests that such management can prevent tourism impact on cave atmospheric particulate matter. This finding is further supported by the lack of high-frequency signals consistent with the passage of tourists in the periodograms of the particulate matter, whereas they clearly indicate, instead, tourism-induced variations on other parameters, most notably CO_2_, temperature and VOCs. In this context, the dynamics of CO_2_ are particularly interesting, since they are considered the most important proxy for the effects of tourism on cave atmosphere (Lobo et al. 2013). Generally, caves have a positive balance of CO_2_, with source processes like decomposition of organic matter, respiration by cave flora and fauna (including tourists), water degassing and diffusion from the above soil largely exceeding the sinks, mainly restricted to water dissolution and fixation by chemolithotrophs (Faimon et al. 2006; de Freitas 2010; Breecker et al. 2012; Mattey et al. 2016). The latter is fostered by nutrient-rich hypogenic waters providing energy to the process (D’Angeli et al. 2019) that, however, lacks in the Pertosa-Auletta Cave and contributes to CO_2_ concentrations topping 2000 ppm in summer-autumn. To a various degree, all the sources and sinks are controlled by temperature and is thus unsurprising the close similarity between the annual dynamics of CO_2_ and temperature in the fossil trail. What is remarkable is the decoupling between these traces in the tourist trail and the substantial overlapping of CO_2_ dynamics between the tourist and the fossil trails, with the former constituting a sort of average of the latter during winter-spring. Here, cave ventilation can play a key role in CO_2_ diffusion, especially in high-energy horizontal caves like the Pertosa-Auletta Cave (Addesso et al. 2019, 2021) facilitating airflow exchanges, as do the inherent seasonality in several processes. Irrespective of the processes involved in shaping the annual trends, however, tourism appears to control CO_2_ dynamics on scales in the order of minutes-few hours only and locally, in the proximity of the visitors. In other words, the monitoring stations are able to record the signals from tourists only when they pass in their proximity, and the signals decay shortly after the passage. This finding holds true also for temperature and, especially, VOCs, contributing to define a comprehensive scenario of tourism-induced alterations to the system. Indeed, when tourists got to the end of the tourist trail, in 2019, the high-frequency oscillations in CO_2_, temperature and VOCs could be recorded by the station in the tourist trail only, with the exception of a faint signal on temperature in the fossil trail. In 2020, when tourists were allowed reaching the “Big room” only and stopping over at around one hundred meters away from the station in the fossil trail, the signals disappeared from the tourist trail and appeared in the fossil trail, albeit fainter and mostly on VOCs only.

The spatial decay of the tourism-induced signals appears thus to be faster for CO_2_ and temperature than for VOCs, and it is likely controlled by the dilution of CO_2_ and VOCs in the cave atmosphere and by the thermal energy absorption as latent heat. In this context, the naturally high CO_2_ concentrations and large thermal capacity of saturated air make up for a quick disappearance of the contributions from tourist breathing and body heat exchanges. The usually low VOC concentrations, instead, allow the emissions from tourists, through exhalation or direct emission from skin and clothes (Fenske and Paulson 1999; Ziwei et al. 2020), to be recorded at larger distances. Interestingly, the absorption of thermal energy inputs as latent heat has been claimed to account for the similar quick decay of thermal signals from tourists also in the Eagle Cave (Domínguez-Villar et al. 2010). Remarkably, short-term tourism-induced variations in temperatures have been recorded in several caves, like the Candamo Cave (Hoyos et al. 1998), the Dechen Cave (Pflitsch et al. 2000), or the Santana Cave (Lobo et al. 2015), with variable recovery rates in the order of hours. This occurrence suggests that the induction of high-frequency temperature transients may be relatively common in show caves, with recoveries controlled by cave characteristics like morphology and hydrology. In this context, the present research introduces a novel dimension to the topic by demonstrating similar dynamics also for CO_2_ and, especially, VOCs that, to our knowledge, has been never adopted in monitoring tourism-induced alterations in underground ecosystems. Incidentally, VOCs is also the parameter showing the clearest responses to tourism, accounting for promising developments in the search for effective proxies of anthropogenic alterations. In terms of temporal scales, the large phase shifts between CO_2_, temperature, VOCs, and the tourist load trace support the hypothesis of alterations dampening shortly after the passage of tourists. Indeed, the latter trace was constructed from the logs of tourist entrances and exits, which made up for unpredictable lags between the alleged and the true passage of visitors in the proximity of the monitoring stations. The phase shifts reflect both these lags and the decay of signals from previously transiting groups, which result in rapid changes in the relative phase of trace oscillations. The time span of these changes allows thus grossly estimating the time scale of the tourism-induced alterations in the order of minutes, which is consistent with spatial scales in the order of few meters. In this context, the similarity in-phase shifts among CO_2_, temperature and VOCs is remarkable and demonstrates the coherent temporal behavior of these proxies of tourism-induced alterations.

## Conclusions

From a conservation perspective, the high-resolution monitoring of the Pertosa-Auletta Cave allowed fulfilling the main goal of the research, i.e., exemplifying a data-driven evaluation of tourism sustainability, but the breadth and implications of the findings are substantially wider. Indeed, they enhance our understanding of the cave ecosystem by shedding light on its dynamics at multiple spatial and temporal scales, on the coupling between the internal and external dynamics, and on the possible drivers of several processes. On top of the relevance these topics bear for the understanding of cave ecosystem ecology, they are crucial in setting references for the evaluation of possible alterations and in defining appropriate conservation measures.

Overall, the tourism-induced alterations of the Pertosa-Auletta Cave integrate within the natural fluctuations by contributing high-frequency signals that decay quickly in space and time. In terms of cave conservation, such alterations are unable to threaten the cave ecosystem functioning under the adopted tourism regimes and demonstrate the sustainability of its management. Although such considerations apply to the Pertosa-Auletta Cave only, due to their dependence upon factors like morphology, climate or hydrogeology, the embraced approach can be straightforwardly adopted into any show cave. Indeed, it is to be hoped that high-resolution monitoring will meet increasingly high adoptions among cave managers, a process that could be only fostered by the low cost of the stations, their adaptability to different requirements through their modularity, and the exemplified analytical flow.

## Supplementary Information

SupplementaryInformation

## Data Availability

Data and materials are available upon request to the authors.

## Code Availability

The code developed for data analysis is available upon request to the authors.
