# Supplementary material for: Underground Ecosystem Conservation Through High-resolution Air Monitoring
Source: Environ Manage. 2022 Feb 21;69(5):982–93. doi: 10.1007/s00267-022-01603-0 (PMC8860376; doi:10.1007/s00267-022-01603-0)
Supplement: Supplementary file 1 — SupplementaryInformation [file 267_2022_1603_MOESM1_ESM.pdf]

Table S1: Main sensors employed in the monitoring stations, with indication of the chip manufacturer and the commercial code.

**Environmental parameter**	**Manufacturer**	**Chip**	**Product code**
T, RH	Sensirion	SHT-10	Adafruit 1298
CO_2	Sensirion	SCD30	SCD30
VOC	Sensirion	SGP30	Adafruit 3709
TPS, PM0.5*, PM1.0, PM2.5, PM4.0, PM10	Sensirion	SPS30	SPS30
Pressure*	NPX Semi	MPL3115A2	Adafruit 1893

*Data recorded but not analyzed in the present research

Table S2: Monthly maxima and minima of all the monitored parameters and the number of tourists.

Trail	Date	T (°C)	RH (%)	CO_2 (ppm)	VOC (ppm)	TPS (μm)	PM1.0 (μg m^{-3})	PM2.5 (μg m^{-3})	PM4.0 (μg m^{-3})	PM10 (μg m^{-3})	Tourists (#)
F	2019 Aug	14.36 – 14.11	100.44 – 96.35	2641 – 1916	694 – 0	1.38 – 0.39	17.65 – 0.79	17.69 – 0.90	17.69 – 0.92	17.69 – 0.92	204 – 0
F	2019 Sep	14.39 – 14.18	100.58 – 100.26	2430 – 2110	453 – 0	1.05 – 0.42	17.59 – 0.41	17.63 – 0.41	17.63 – 0.41	17.63 – 0.41	273 – 0
F	2019 Oct	14.32 – 14.02	100.57 – 100.30	2524 – 1987	552 – 0	0.87 – 0.42	154.03 – 0.02	162.05 – 0.02	163.07 – 0.02	163.24 – 0.02	197 – 0
F	2019 Nov	14.20 – 13.84	100.61 – 100.15	2494 – 1800	569 – 0	0.87 – 0.48	95.32 – 2.23	95.50 – 2.26	95.50 – 2.27	95.51 – 2.27	212 – 0
F	2019 Dec	13.92 – 13.44	100.32 – 99.67	2121 – 1800	409 – 0	0.78 – 0.49	234.78 – 8.47	235.23 – 8.49	235.24 – 8.5	235.24 – 8.50	164 – 0
F	2020 Jan	13.54 – 13.28	99.90 – 95.24	1221 – 591	8464 – 0	0.71 – 0.50	272.62 – 13.12	273.59 – 13.14	273.66 – 13.15	273.68 – 13.15	130 – 0
F	2020 Feb	13.51 – 13.24	98.92 – 98.16	1524 – 562	1354 – 0	0.81 – 0.48	144.87 – 5.28	145.14 – 5.29	145.15 – 5.29	145.15 – 5.29	158 – 0
F	2020 Mar	13.42 – 13.19	99.27 – 98.60	1570 – 545	866 – 0	0.82 – 0.46	204.53 – 5.8	204.92 – 5.83	204.93 – 5.84	204.93 – 5.84	31 – 0
F	2020 Apr	13.50 – 13.20	99.70 – 98.70	1508 – 561	870 – 0	0.66 – 0.49	240.67 – 5.08	241.12 – 5.09	241.13 – 5.09	241.14 – 5.09	0 – 0
F	2020 May	13.80 – 13.42	100.42 – 99.37	1444 – 793	513 – 0	0.69 – 0.43	47.35 – 0.96	47.44 – 0.96	47.44 – 0.96	47.44 – 0.96	0 – 0
F	2020 Jun	13.93 – 13.60	100.62 – 99.77	2650 – 1188	290 – 0	0.71 – 0.40	34.29 – 0.31	34.36 – 0.31	34.36 – 0.31	34.36 – 0.31	0 – 0
F	2020 Jul	14.14 – 13.86	101.00 – 100.53	2753 – 2099	568 – 0	0.71 – 0.37	228.66 – 0.02	229.09 – 0.02	229.10 – 0.02	229.10 – 0.02	76 – 0
F	2020 Aug	14.46 – 14.08	101.41 – 100.96	2624 – 2120	2507 – 0	1.11 – 0.37	17.59 – 0.02	17.63 – 0.03	17.63 – 0.03	17.63 – 0.03	99 – 0
F	2020 Sep	14.80 – 14.32	101.43 – 101.15	2581 – 1657	1817 – 0	0.97 – 0.42	55.22 – 0.48	55.33 – 0.50	55.33 – 0.50	55.33 – 0.50	103 – 0
T	2019 Aug	15.00 – 14.63	97.91 – 93.76	2376 – 2038	3139 – 0	1.63 – 0.60	159.99 – 0.00	179.71 – 0.00	182.23 – 0.00	182.64 – 0.00	204 – 0
T	2019 Sep	14.97 – 14.62	98.33 – 97.20	2521 – 2147	7057 – 0	0.99 – 0.37	22.92 – 0.00	24.93 – 0.00	25.18 – 0.00	25.23 – 0.00	273 – 0
T	2019 Oct	14.84 – 14.62	98.93 – 97.59	2565 – 2107	11821 – 0	1.42 – 0.34	30.92 – 0.00	39.39 – 0.00	40.48 – 0.00	40.66 – 0.00	197 – 0
T	2019 Nov	15.02 – 14.64	99.15 – 97.76	2690 – 1784	9006 – 0	1.45 – 0.35	20.44 – 0.00	25.91 – 0.00	26.61 – 0.00	26.72 – 0.00	212 – 0
T	2019 Dec	15.19 – 14.73	99.82 – 98.24	2158 – 1784	6887 – 0	1.15 – 0.38	55.27 – 0.00	66.18 – 0.00	67.59 – 0.00	67.81 – 0.00	164 – 0
T	2020 Jan	15.06 – 14.82	99.85 – 96.62	1016 – 582	12249 – 0	0.91 – 0.40	99.79 – 6.12	99.99 – 6.13	99.99 – 6.13	99.99 – 6.13	130 – 0
T	2020 Feb	15.03 – 14.79	99.99 – 98.68	1211 – 526	17987 – 0	1.13 – 0.39	56.65 – 0.76	56.76 – 0.76	56.76 – 0.76	56.77 – 0.76	158 – 0
T	2020 Mar	14.94 – 14.76	100.21 – 99.01	1273 – 514	10640 – 0	0.69 – 0.40	48.53 – 0.00	48.63 – 0.00	48.63 – 0.00	48.63 – 0.00	31 – 0
T	2020 Apr	14.92 – 14.77	100.25 – 99.23	1260 – 550	1340 – 0	0.76 – 0.37	56.64 – 0.00	56.76 – 0.00	56.76 – 0.00	56.76 – 0.00	0 – 0
T	2020 May	14.87 – 14.67	100.00 – 99.25	1651 – 940	672 – 0	1.50 – 0.33	9.33 – 0.00	9.35 – 0.00	9.35 – 0.00	9.35 – 0.00	0 – 0
T	2020 Jun	14.83 – 14.62	99.68 – 99.20	2024 – 1284	4283 – 0	1.24 – 0.32	6.61 – 0.00	7.62 – 0.00	7.75 – 0.00	7.77 – 0.00	0 – 0
T	2020 Jul	14.77 – 14.55	99.62 – 99.09	2381 – 1861	9119 – 0	1.38 – 0.31	8.23 – 0.00	8.24 – 0.00	8.24 – 0.00	8.24 – 0.00	76 – 0
T	2020 Aug	14.96 – 14.56	99.88 – 99.21	2446 – 2014	13471 – 0	1.13 – 0.32	8.9 – 0.00	8.92 – 0.00	8.92 – 0.00	8.92 – 0.00	99 – 0
T	2020 Sep	16.15 – 14.78	100.26 – 99.08	2781 – 2010	1900 – 0	1.27 – 0.34	13.35 – 0.00	13.37 – 0.00	13.37 – 0.00	13.37 – 0.00	103 – 0

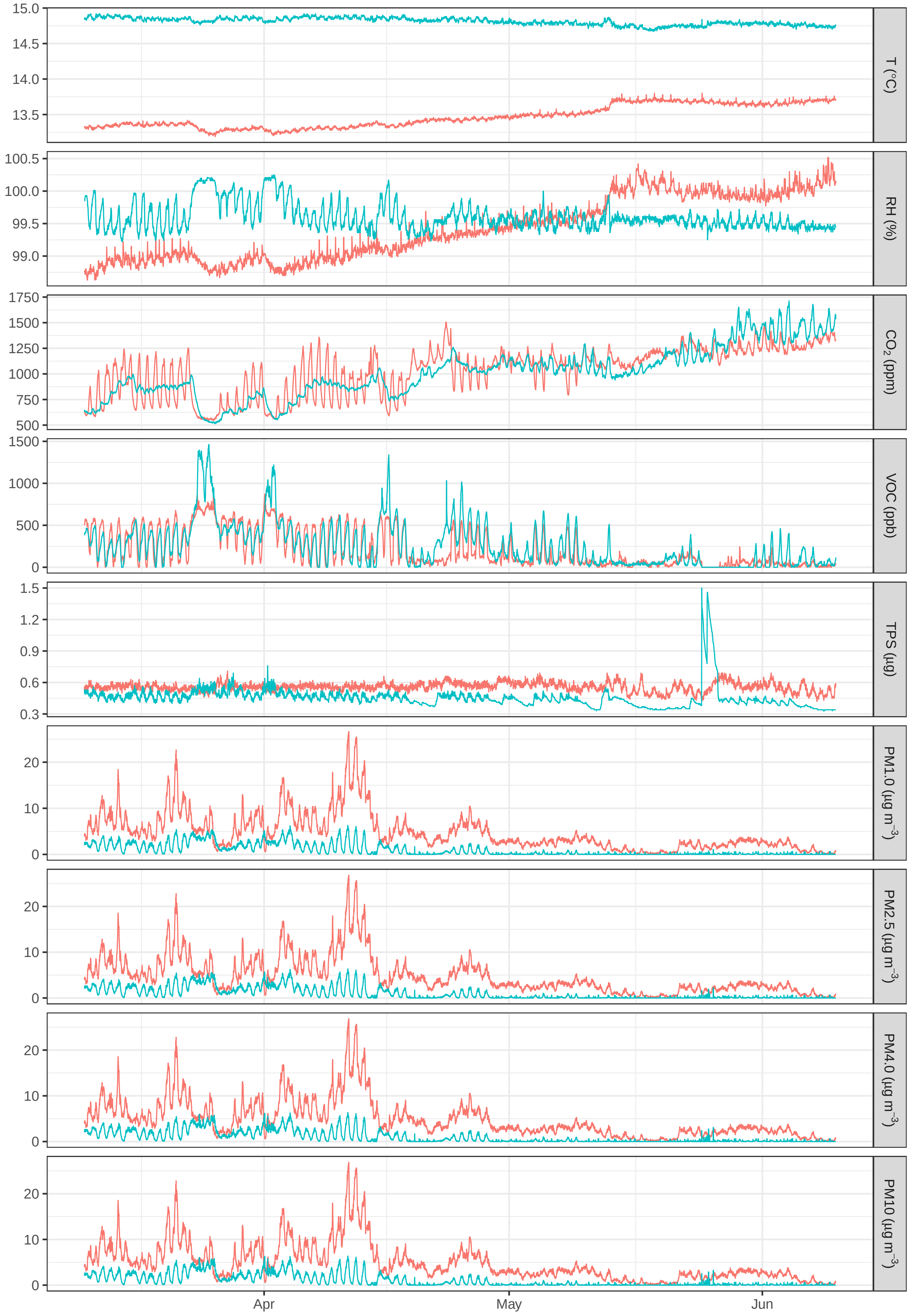

Fig. S1: Extract from the time series of all the parameters monitored (temperature: T, relative humidity: RH, CO_2, VOC, typical particulate size: TPS, PM1.0, PM2.5, PM4.0, PM10) during the lockdown phase (from 2020-03-10 00:00:00 to 2020-06-10 00:00:00) in the tourist (green) and fossil (red) trails.

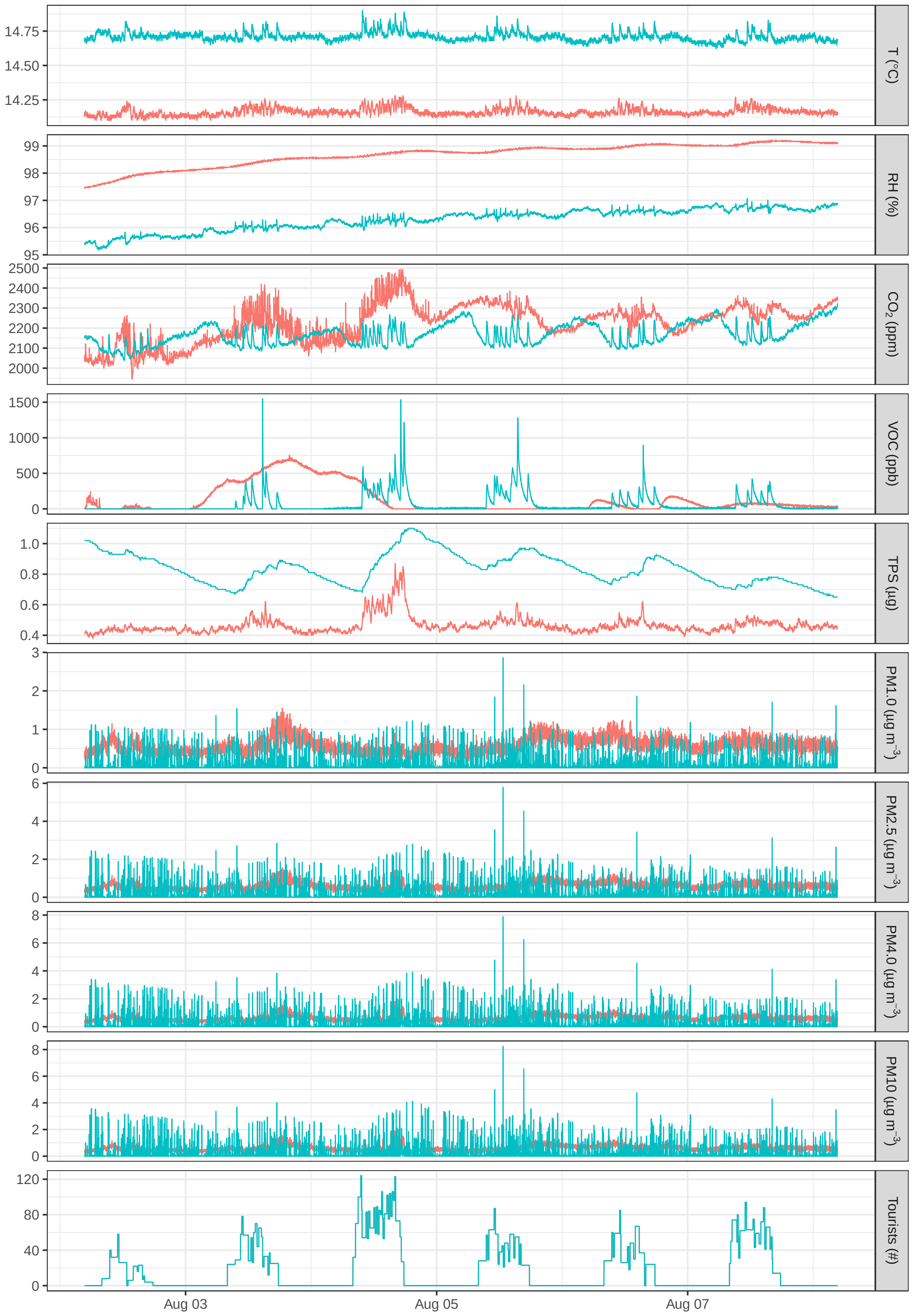

Fig. S2: Extract from the time series of all the parameters monitored (temperature: T, relative humidity: RH, CO_2, VOC, typical particulate size: TPS, PM1.0, PM2.5, PM4.0, PM10) and of the number of tourists during the first week of August 2019 (from 2019-08-02 04:41:00 to 2019-08-08 04:41:00) in the tourist (green) and fossil (red) trails.

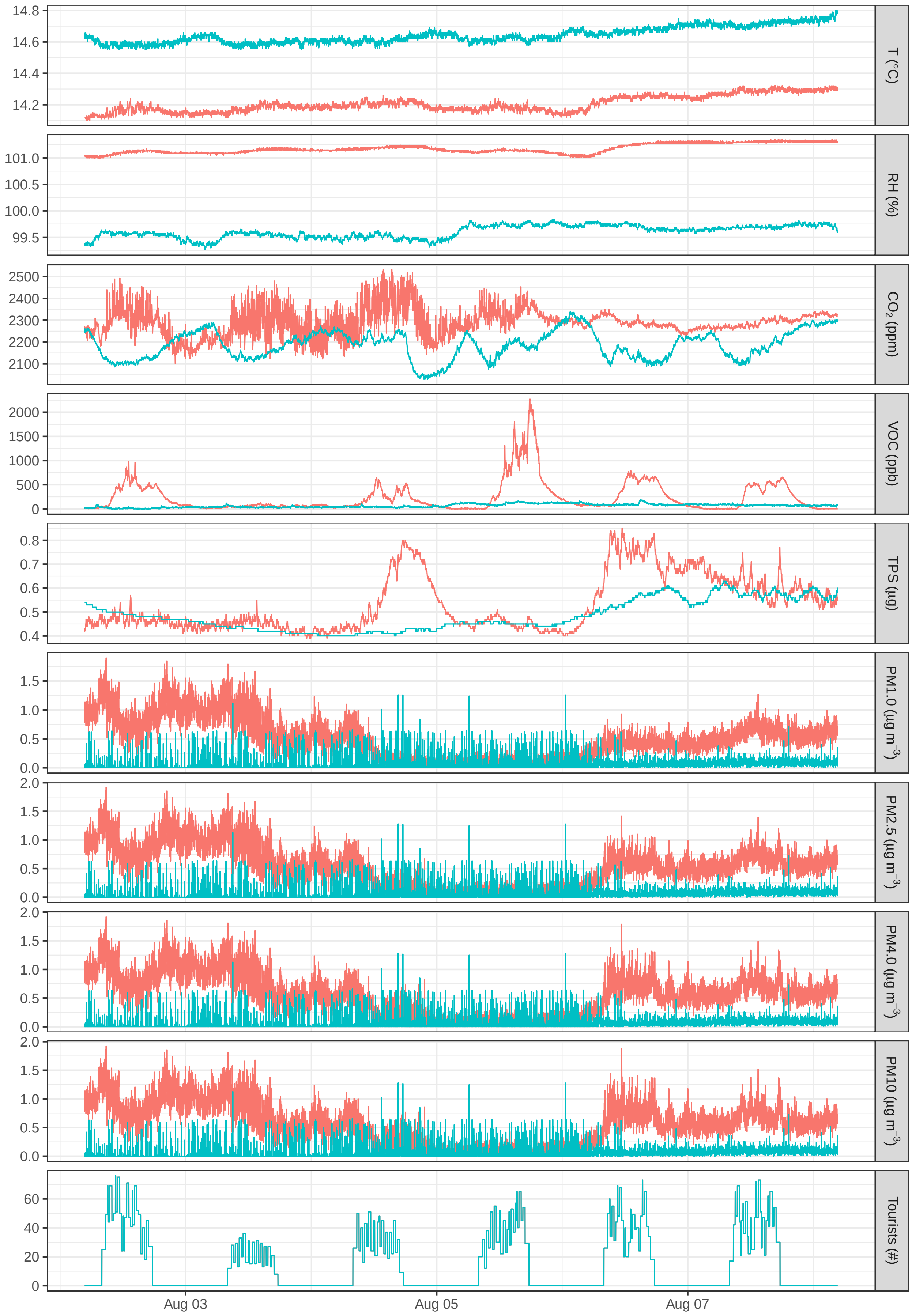

Fig. S3: Extract from the time series of all the parameters monitored (temperature: T, relative humidity: RH, CO_2, VOC, typical particulate size: TPS, PM1.0, PM2.5, PM4.0, PM10) and of the number of tourists during the first week of August 2020 (from 2020-08-02 04:41:00 to 2020-08-08 04:41:00) in the tourist (green) and fossil (red) trails.
